# Effectiveness of enteral feeding protocol on clinical outcomes in critically ill patients: A before and after study

**DOI:** 10.1371/journal.pone.0182393

**Published:** 2017-08-03

**Authors:** Qian Li, Zhongheng Zhang, Bo Xie, Xiaowei Ji, Jiahong Lu, Ronglin Jiang, Shu Lei, Shihao Mao, Lijun Ying, Di Lu, Xiaoshui Si, Mingxia Ji, Jianxing He, Mengyan Chen, Wenjuan Zheng, Jiao Wang, Jing Huang, Junfeng Wang, Yaling Ji, Guodong Chen, Jianhua Zhu, Yadi Shao, Ronghai Lin, Chao Zhang, Weiwen Zhang, Jian Luo, Tianzheng Lou, Xuwei He, Kun Chen, Wei Peng, Renhua Sun

**Affiliations:** 1 Department of Critical Care Medicine, Zhejiang Provincial People's Hospital, Zhejiang, P. R. China; 2 Department of emergency medicine, Sir Run-Run Shaw Hospital, Zhejiang University School of Medicine, Hangzhou, China; 3 Department of Critical Care Medicine, Huzhou Central Hospital, Zhejiang, China; 4 Department of Critical Care Medicine, Zhejiang Provincial Hospital of TCM, Zhejiang, China; 5 Department of Critical Care Medicine, Shaoxing People's Hospital, Zhejiang, China; 6 Department of Critical Care Medicine, YiWu Central Hospital, Zhejiang, P. R. China; 7 Department of Critical Care Medicine, NingBo First Hospital, Zhejiang, China; 8 Department of Critical Care Medicine, TaiZhou Hospital, Zhejiang, China; 9 Department of Critical Care Medicine, QuZhou People's Hospital, Zhejiang, P. R. China; 10 Department of Critical Care Medicine, LiShui People's Hospital, Zhejiang, P. R. China; 11 Department of Critical Care Medicine, Jinhua Municipal Central Hospital, Jinhua Hospital of Zhejiang University, Zhejiang, P. R. China; University of Florida, UNITED STATES

## Abstract

**Background and objective:**

Enteral nutrition (EN) feeding protocol was proposed to have positive impact on critically ill patients. However, current studies showed conflicting results. The present study aimed to investigate whether enteral feeding protocol was able to improve clinical outcomes in critically ill patients.

**Methods:**

A before (stage 1) and after (stage 2) interventional study was performed in 10 tertiary care hospitals. All patients expected to stay in the intensive care unit (ICU) for over three days were potentially eligible. Clinical outcomes such as 28-day mortality, ICU length of stay, duration of mechanical ventilation (MV), and nosocomial infection were compared between the two stages.

**Main results:**

A total of 410 patients were enrolled during the study period, including 236 in stage 1 and 174 in stage 2. EN feeding protocol was able to increase the proportion of EN in day 2 (41.8±22.3 vs. 50.0±28.3%; p = 0.006) and day 6 (70.3±25.2 vs. 77.6±25.8%; p = 0.006). EN percentages tended to be higher in stage 1 than that in stage 2 on other days, but statistical significance was not reached. There was no difference in 28-day mortality between stage 1 and 2 (0.14 vs. 0.14; p = 0.984). Implementation of EN feeding protocol marginally reduced ICU length of stay (19.44±18.48 vs. 16.29±16.19 days; p = 0.077). There was no difference in the duration of MV between stage a and stage 2 (14.24±14.49 vs. 14.51±17.55 days; p = 0.877).

**Conclusions:**

The study found that the EN feeding protocol was able to increase the proportion of EN feeding, but failed to reduce 28-day mortality, incidence of nosocomial infection or duration of MV.

## Introduction

Critically ill patients are at increased risk of death, and they are usually treated at intensive care unit (ICU) [1]. Because critical illness typically involves multiple organs, multidisciplinary approaches are required for the management of them. Among all interventions, nutrition therapy is one of the most important interventions that may significantly influence the clinical outcomes [2,3]. There is a large body of evidence showing that malnutrition is associated with significantly increased risk of death [4,5]. ICU patients are at increased risk of underfeeding, further exacerbating the existing gap between energy demand and intake [6].

The route of nutrition delivery is another important issue when starting nutrition therapy for ICU patients. It is widely accepted that enteral nutrition (EN) is better than parenteral nutrition [7,8]. There is a variety of reasons that may delay the administration of EN in ICU, which include but not limited to, recent abdominal surgery, hemodynamic instability, physicians’ unawareness of the importance of EN, large gastric residual volume (GVR) and gastrointestinal abnormality [9]. While some of the reasons are real contraindications of EN, others (such as GRV) may be lack of evidence that they are real contraindications [10]. Because nutrition therapy involves a battery of interventions and procedures, a variety of clinical practice guidelines have been published to standardize EN delivery [11,12]. However, it was reported that the adherence to these guidelines were suboptimal. Furthermore, the effect of the implementation of enteral feeding protocol on clinical outcomes remains controversial [13]. For example, Declercq B and colleagues reported that the implementation of enhanced protein-energy provision appeared ineffective in improving nutritional intake in surgical ICU patients [14]. Other studies showed that implementing nutrition support algorithm could help to improve delivery of nutrients [15–18]. However, it remains controversial on whether enteral feeding protocol is effective in improving patient important outcomes such as mortality, nosocomial infections, duration of mechanical ventilation and length of stay in ICU [19–22]. The aim of the present study was to investigate whether enteral feeding protocol would improve clinical outcomes for critically ill patients.

## Methods

### Study population

The study protocol was published elsewhere [23], and here we describe it briefly. The study was conducted in 10 tertiary care hospitals. All patients expected to stay in ICU for over three days were potentially eligible. Exclusion criteria included: 1) Contraindications to enteral feeding such as mechanical bowel obstruction, shock requiring high-dose vasopressors, massive gastrointestinal bleeding, severe abdominal infection, persistent paralytic ileus, acute phase of short bowel syndrome, acute phase (less than month) of extensive small bowel resection, jejunal fistula, refractory diarrhea, persistent severe vomiting, severe inflammatory bowel disease, acute phase of severe pancreatitis; 2) subjects receiving EN in previous 7 days; 3) contraindications for nasogastric or nasoenteric tube placement; 4) subjects who had already undergone percutaneous endoscopic jejunostomy (PEJ), percutaneous endoscopic gastrostomy (PEG) and surgical jejunostomy; 5) age younger than 18 years old; 6) women who are pregnant or undergo breast feeding; 7) burn patients [23].

### Study design

The study was conducted from April 2016 to January 2017, and included two stages. Stage 1 lasted from April 2016 to July 2016, and stage 2 lasted from September 2016 to January 2017. There was a training period from August 2016 to September 2016. During stage 1, the attending physicians were allowed to deliver EN under their discretions or according to local policies. During the training period, all physicians, nurses and dieticians from participating centers were trained by using standardized enteral nutrition feeding protocol. We initially planned a two-week training program. However, the training quality was considered to be undesirable due to the short training period. Thus, we extended the training period to two months, ensuring all participants can master the standardized EN feeding protocol. Stage 2 was a period during which the standardized enteral feeding protocol was fully implemented in all participating centers. The compliance to the protocol was monitored and promoted by a designated investigator in each center.

The study was approved by the ethics committee of Zhejiang provincial people’s hospital (approval No. 2016JS001), and was registered at International Standard Registered Clinical/soCial sTudy Number (ISRCTN) registry (ISRCTN10583582). Patients’ information was de-identified after data collection. Informed consent was obtained from the patients or their relatives. The study was reported according to the STROBE checklist (S1 Table). The dataset was available at the supplemental file (S2 Table)

### Enteral feeding protocol

**Fig 1:**
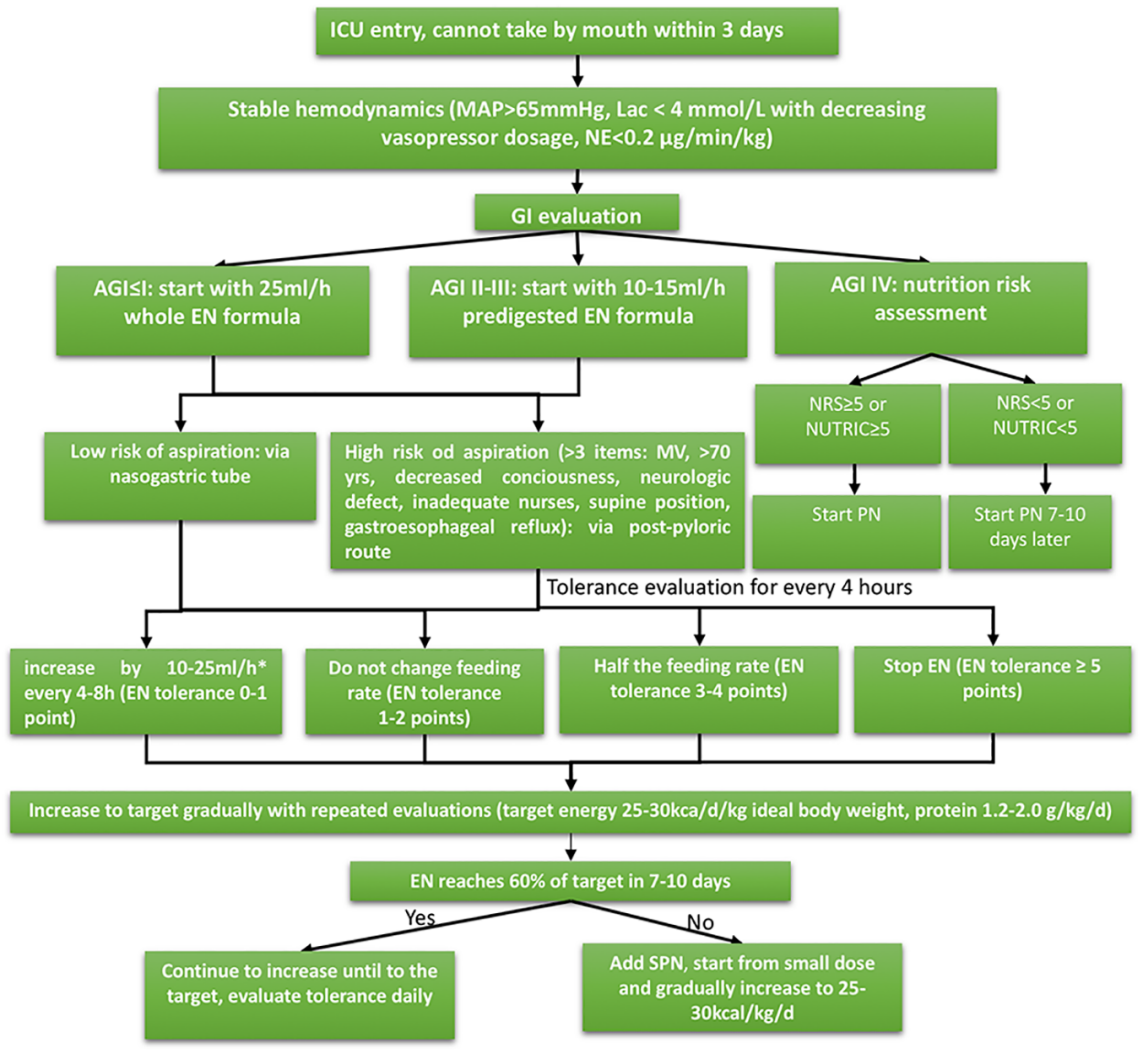
Enteral feeding algorithm. Before EN initiation, Hemodynamic should be stabilized with MAP>65 mmHg and lactate<4 mmol/l, or vasopressor dose was decreasing. GI function was then evaluated with the AGI staging system. For pateints with AGI of I, EN could be started at 25 ml/h. For patients with AGI II-III, predigested EN could be started at 10–15 ml/h. EN was withheld for those with AGI IV. If patients were at high risk of malnutrition, parenteral nutrition (PN) should started.

The enteral feeding protocol was modified and slightly different from its original version as described in the previous publication [23]. Herein we describe the most updated enteral feeding protocol. Fig 1 shows the enteral feeding algorithm. Before EN initiation, Hemodynamic should be stabilized with MAP>65 mmHg and lactate<4 mmol/l, with decreasing vasopressor dose. Gastrointestinal function was then evaluated with the acute gastrointestinal injury (AGI) grading system. For patients with AGI of I, EN was started at 25 ml/h. For patients with AGI II-III, predigested EN was started at 10–15 ml/h. EN was withheld for those with AGI IV. If patients were at high risk of malnutrition, parenteral nutrition (PN) should be started. Otherwise, PN was withheld for 7–10 days. Patients on EN would be evaluated using tolerance score for every 4 hours. The tolerance score is shown in Table 1. EN was discontinued when EN tolerance score was greater than 5 points.

**Table 1:** Enteral nutrition tolerance score.

Points	0	1	2	5
Abdominal distension/pain	None	Mild distension; No distension	Moderate distension; Spontaneous resolution of abdominal pain; IAP: 15~20mmHg	Severe distension; No resolution of abdominal pain; IAP>20mmHg
Nausea/vomiting	None; continuous gastric decompression without symptom	Nausea but no vomiting	Nausea and vomiting without need for decompression or GRV>250 ml/l	Vomiting requiring gastric decompression or GRV>500 ml/l
Diarrhea	None	Loose stools with volume <250 ml every 4 hour	Loose stools once or twice per 4h with volumes 250–500ml	Loose stools > twice per 4h; Volume >500ml

**Fig 2:**
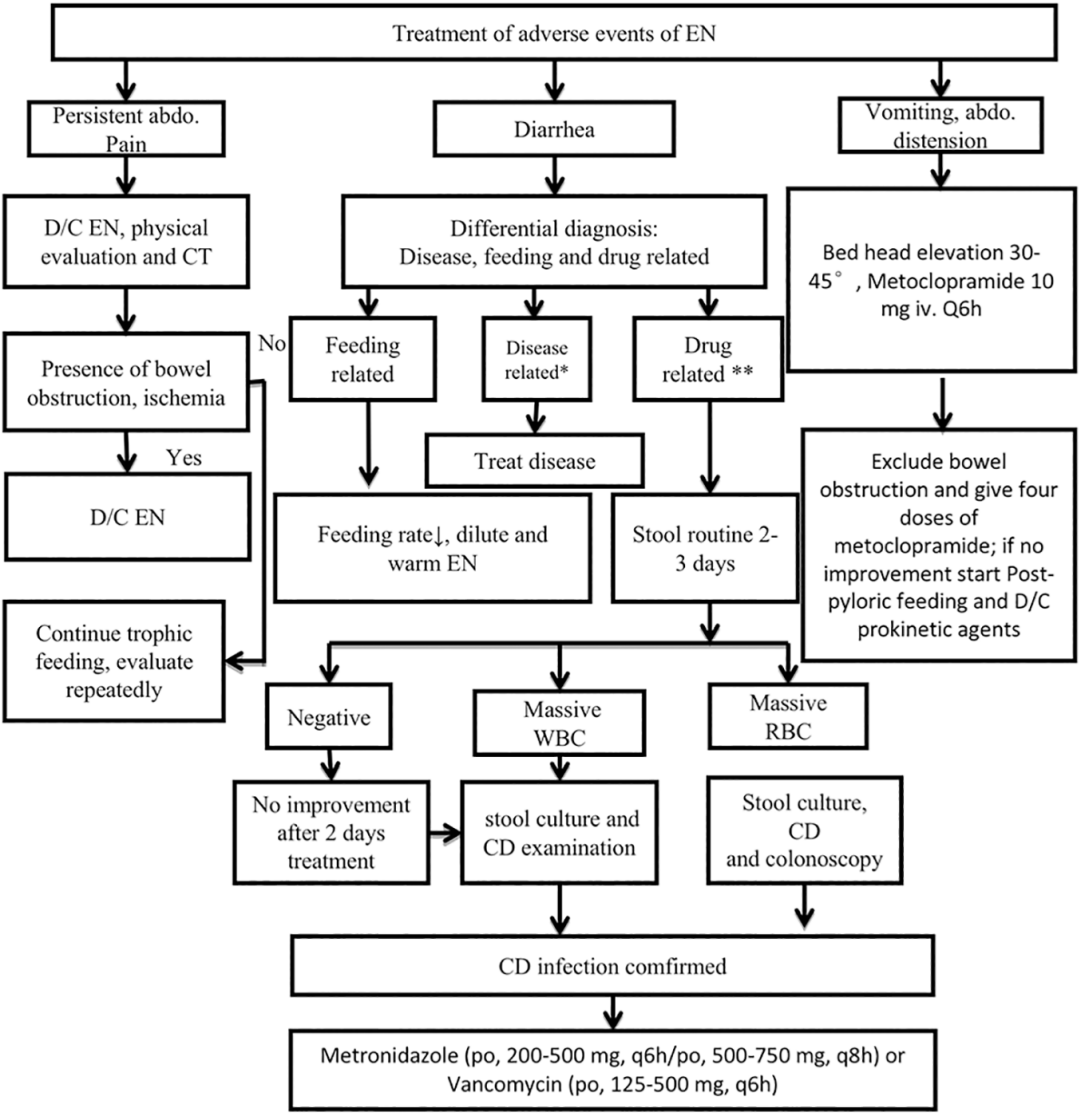
Protocols for the management of adverse events. EN was discontinued if persistent abdominal pain occurred. Physical examination and abdominal computed tomography would be ordered. If there was bowel obstruction or ischemia, EN would be discontinued. Diarrhea could be caused by enteral feeding, specific diseases and drugs. If clostridium difficile (CD) infection was identified, the patient should be treated with metronidazole or vancomycin. If the patient experienced vomiting and/or abdominal distension, bed head should be elevated to 30 to 40 degrees with administration of metoclopramide.

Adverse events were treated and managed with standardized protocol (Fig 2). EN was discontinued in the presence of persistent abdominal pain. Physical examination and abdominal computed tomography would be ordered. If there was bowel obstruction and/or ischemia, EN should be discontinued. Diarrhea could be caused by enteral feeding, specific diseases and drugs, and infections. If clostridium difficile (CD) infection was identified, the patient was treated with metronidazole or vancomycin. If the patient experienced vomiting and/or abdominal distension, bed head should be elevated to 30 to 45 degrees with administration of metoclopramide.

### Outcomes

The primary outcome was the 28-day mortality, which was defined as the vital status at 28 days after enrollment. Secondary outcomes included duration of mechanical ventilation, ICU length of stay, nosocomial infection. Nosocomial infection was defined as any infections occurred 48 hours after admission.

### Statistical analysis

Continuous variables were described as mean and standard deviation, and were compared between stage 1 and 2 by t test. Categorical variables were described as the number and proportion, and were compared using Chi-square or Fisher exact test as appropriate [24]. Missing values were unavoidable in data entry. Missing categorical variables were imputed with mode value of that variable, and missing continuous variable were imputed with the mean value of that variable [25,26]. The primary outcome was vital status at 28 days after enrollment, which was compared between stage 1 and 2. Other secondary outcome variables such as ICU discharge status, nosocomial infections, ICU length of stay and duration of MV were compared between patients enrolled in stage 1 and 2. Because this was not a randomized controlled trial, it was expected to have imbalance in baseline characteristics between stage 1 and 2 [27–29], thus we performed multivariable Logistic regression analysis [30]. All variables with a p value less than 0.2 in univariate analysis were entered into the model. The stage variable was forced into the model. We added an interaction term between age and stage to the model, assuming that the impact of enteral feeding protocol had different impact on old and young patients.

All statistical analyses were performed using R (version 3.3.2), a two-tailed p value less than 0.05 was considered to be statistically significant.

## Results

**Fig 3:**
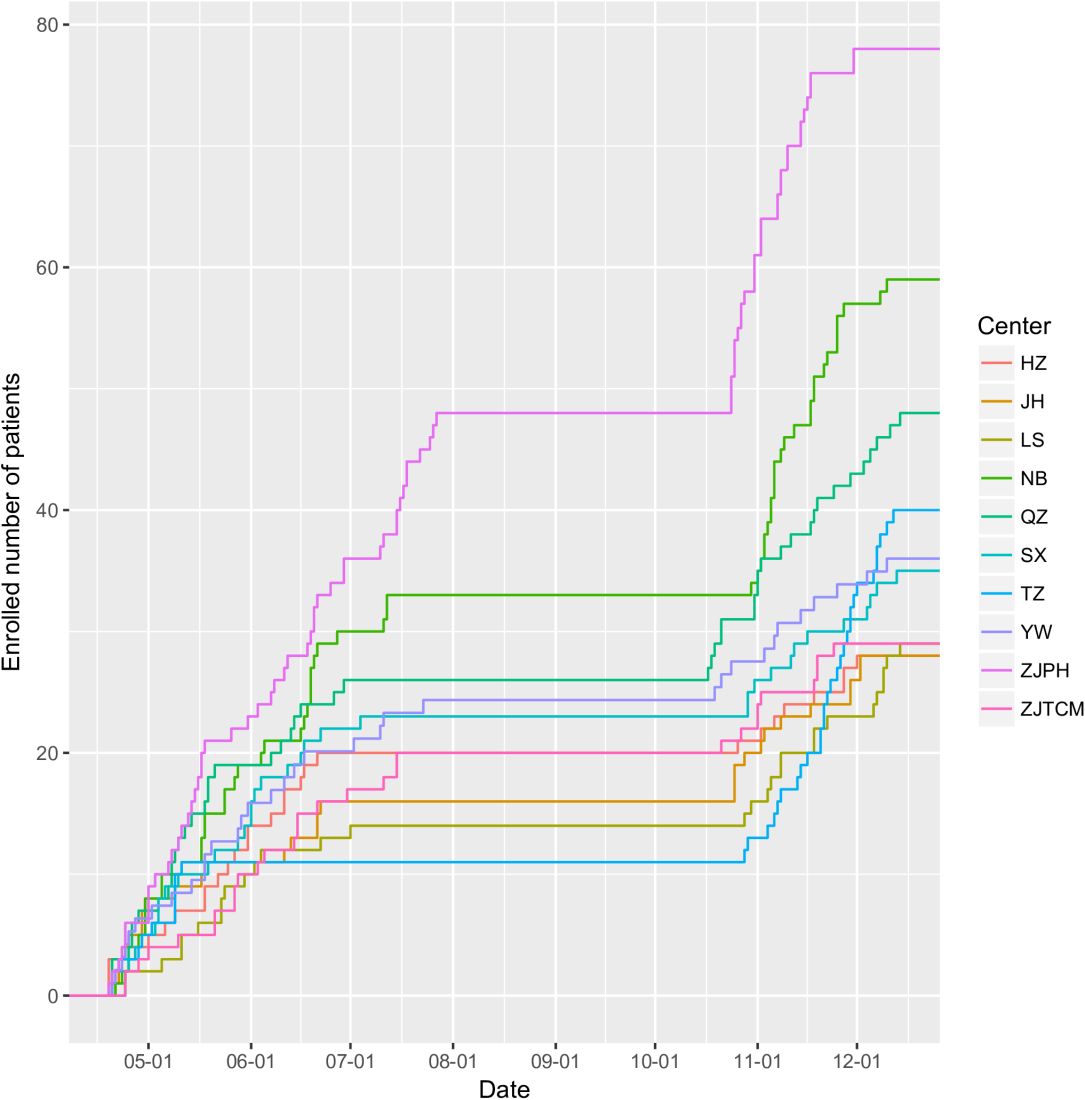
Subject enrollment in each participating center over the study period.

**Fig 4:**
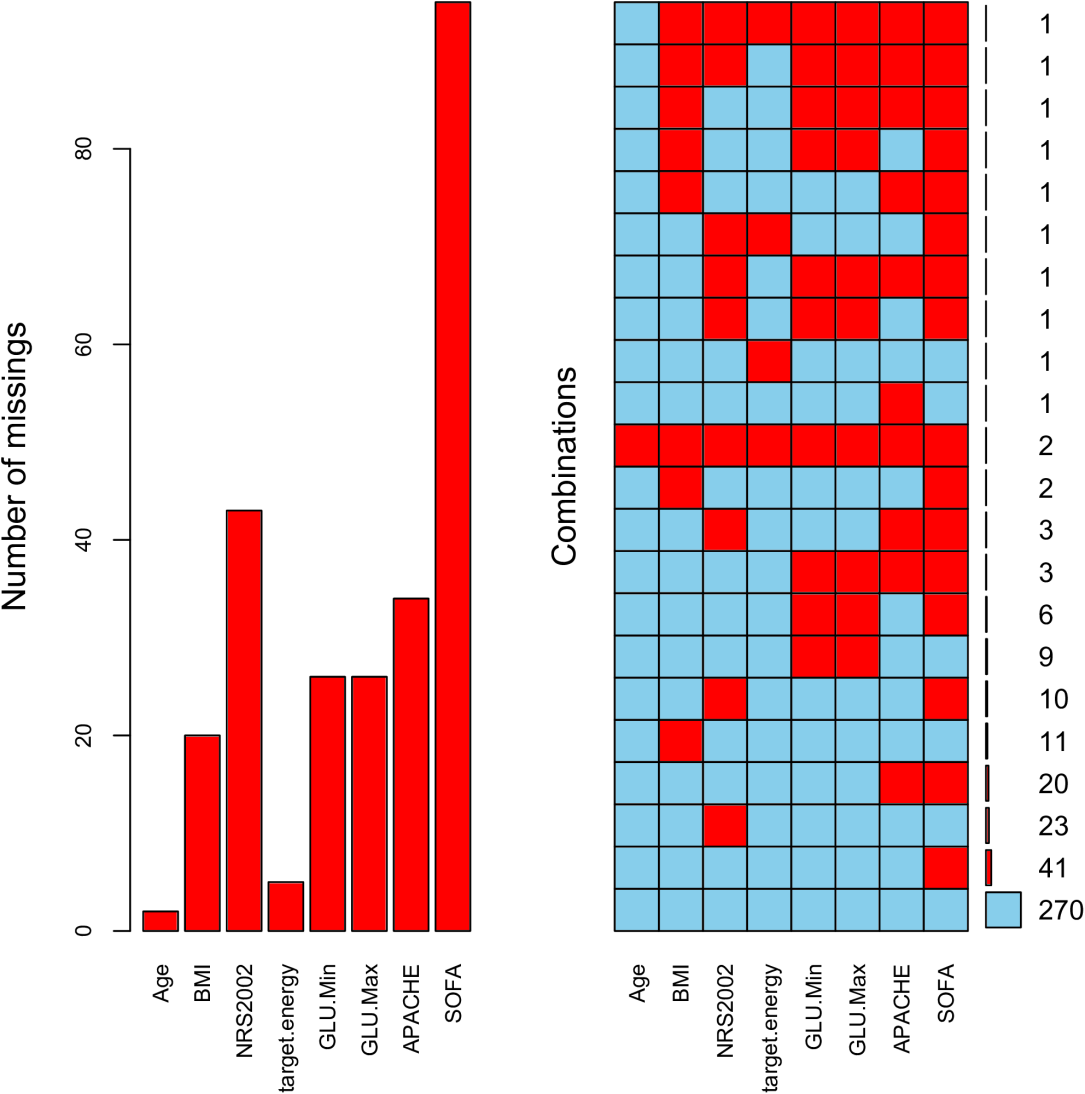
Missing pattern of some important variables.

A total of 410 patients were enrolled in the study, including 236 in stage 1 and 174 in stage 2. Fig 3 shows the subject enrollment in each participating center. Note there was a flat portion between August and September, which was the training period. There were missing values in the study (Fig 4). Baseline characteristics of patients enrolled in stage 1 and 2 are shown in Tables 2 and 3. Patients in stage 1 were significantly elder (65.01±17.13 vs. 64.30±16.70; p = 0.033), had higher NRS scores (3.71±1.02 vs. 3.60±0.91; p = 0.013) than patients in stage 2. The GI function was better in stage 1 patients, as represented by more number of patients with AGI-I (0.73 vs. 0.59; p = 0.007) and less patients with AGI-III (0.06 vs. 0.13; p = 0.017). The use of PN was significantly reduced in stage 2 as compared with that in stage 1 (0.25 vs. 0.09; p<0.01).

**Table 2:** Comparisons of baseline characteristics of subjects enrolled in the two stages (continuous variables).

	Mean (overall)	SD (overall)	Mean (stage 1)	SD (stage 1)	Mean (stage 2)	SD (stage 2)	p
Age (years)	64.71	16.93	65.01	17.13	64.30	16.70	0.033
BMI (kg/m^2^)	22.75	3.64	23.02	3.37	22.39	3.96	0.262
NRS2002	3.67	0.97	3.71	1.02	3.60	0.91	0.013
Energy goal (kcal)	1632.18	340.64	1691.93	374.62	1551.25	268.87	0.154
EN proportion*	33.49	22.61	31.57	22.18	36.56	23.08	0.176
Minimum glucose	7.29	2.46	7.33	2.71	7.25	2.10	0.952
Maximum glucose	11.55	3.84	11.36	4.18	11.80	3.35	0.151
APACHEII	18.58	7.44	18.48	7.16	18.71	7.85	0.233
SOFA	5.90	3.63	5.63	3.39	6.34	3.95	0.277

Abbreviations: BMI: body mass index; NRS: nutrition risk score; EN: enteral nutrition; APACHEII: Acute Physiology and Chronic Health Evaluation II; SOFA: sequential organ failure assessment.

Note:

* EN proportion refers the the proportion of energy intake administered via gastrointestinal tract.

**Table 3:** Comparisons of baseline characteristics of subjects enrolled in the two stages (categorical variables).

	Number (overall)	Proportion (overall)	Number (stage 1)	Proportion (stage 1)	Number (stage 2)	Proportion (stage 2)	p
Sex (male)	263	0.64	158	0.67	105	0.60	0.155
Source							
Ward	285	0.70	158	0.67	127	0.73	0.228
Post-operation	34	0.08	22	0.09	12	0.07	0.485
Emergency	101	0.25	67	0.28	34	0.20	0.052
Sepsis	50	0.12	29	0.12	21	0.12	1.000
AKI	73	0.18	48	0.20	25	0.14	0.152
Glucocorticoid	54	0.13	34	0.14	20	0.11	0.475
Malnutrition	182	0.44	105	0.44	77	0.44	1.000
Vasopressors	135	0.33	83	0.35	52	0.30	0.308
MV	352	0.86	201	0.85	151	0.87	0.749
AGI.I	276	0.67	173	0.73	103	0.59	0.007
AGI.II	67	0.16	41	0.17	26	0.15	0.601
AGI.III	35	0.09	13	0.06	22	0.13	0.017
AGI.IV	2	0.00	1	0.00	1	0.01	1.000
Enteral nutrition use	173	0.42	100	0.42	73	0.42	1.000
Parenteral nutrition	74	0.18	59	0.25	15	0.09	<0.01

Abbreviations: AKI: acute kidney injury; MV: mechanical ventilation; AGI: acute gastrointestinal injury.

Table 4 shows the primary and secondary outcomes. There was no difference in 28-day mortality between stage 1 and 2 (0.14 vs. 0.14; p = 0.984). The ICU discharge status was marginally significant, with more proportion of patients transferred to the ward in stage 1 than stage 2 (0.45 vs. 0.37; p = 0.065). Nosocomial infection was not significantly different between the two stages (0.20 vs. 0.16; p = 0.406). Implementation of EN feeding protocol marginally reduced ICU length of stay (19.44±18.48 vs. 16.29±16.19 days; p = 0.077). Duration of MV was not significantly different between the two stages (14.24±14.49 vs. 14.51±17.55 days; p = 0.877).

**Table 4:** Comparisons of outcome variables of stage one versus stage two.

Outcome variables	Overall	Stage 1	Stage 2	p
The primary outcome				
28-day mortality	57 (0.14)	33 (0.14)	24 (0.14)	0.984
Secondary outcomes				
ICU discharge status				0.065
AAD	69 (0.17)	41 (0.17)	28 (0.16)	
Die	41 (0.10)	24 (0.10)	17 (0.10)	
Discharge home	106 (0.26)	61 (0.26)	45 (0.26)	
Transfer to ward	166 (0.40)	87 (0.37)	79 (0.45)	
Nosocomial infection	73 (0.18)	38 (0.16)	35 (0.20)	0.406
ICU length of stay	18.05±17.55	19.44±18.48	16.29±16.19	0.077
Duration of MV	14.37±15.95	14.24±14.49	14.51±17.55	0.877

Abbreviations: AAD: against advice discharge; ICU: intensive care unit; MV: mechanical ventilation.

**Fig 5:**
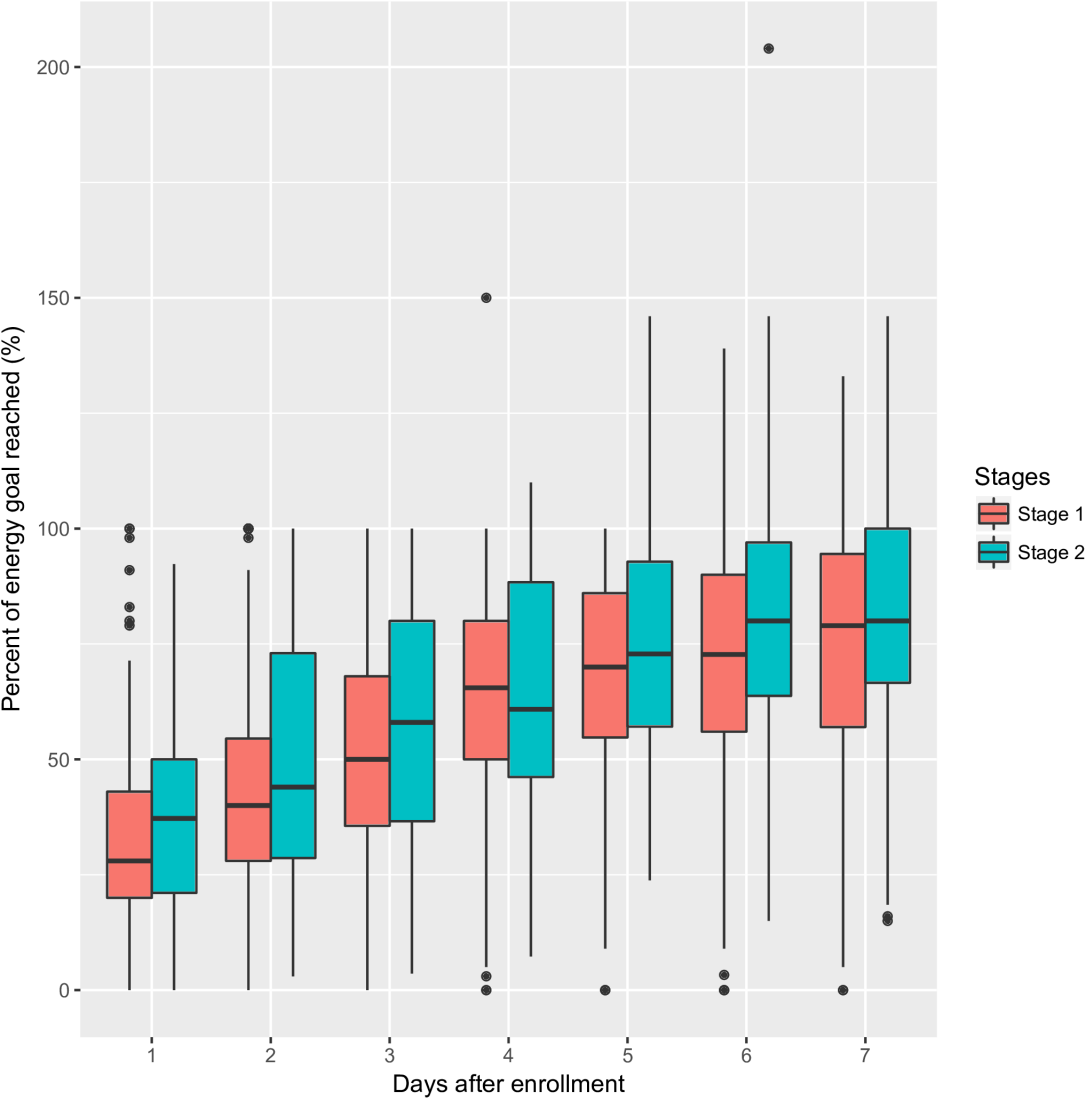
Comparisons of EN proportion between stage 1 and 2. Patients in stage 2 received more EN than that in stage 1. The proportion of EN increased from day 1 to day 7.

EN feeding protocol was able to increase the proportion of EN in day 2 (41.8±22.3 vs. 50.0±28.3%; p = 0.006) and day 6 (70.3±25.2 vs. 77.6±25.8%; p = 0.006). EN percentages were higher on other days, but statistical significance was not reached (Table 5). Also, EN proportions were increased from day 1 to day 7 (Fig 5).

**Table 5:** Comparisons of the percentage (%) of estimated energy goal reached from day 1 to day 7.

	Mean (stage 1)	SD (stage 1)	Mean (stage 2)	SD (stage 2)	p
Day 1	31.9	22.5	36.6	23.1	0.156
Day 2	41.8	22.3	50.0	28.3	0.006
Day 3	53.7	23.3	58.5	25.6	0.074
Day 4	63.6	23.5	65.2	24.5	0.542
Day 5	68.9	23.7	72.8	22.8	0.135
Day 6	70.3	25.2	77.6	25.8	0.012
Day 7	73.4	24.2	77.9	24.0	0.122

Abbreviations: SD: standard deviation.

**Fig 6:**
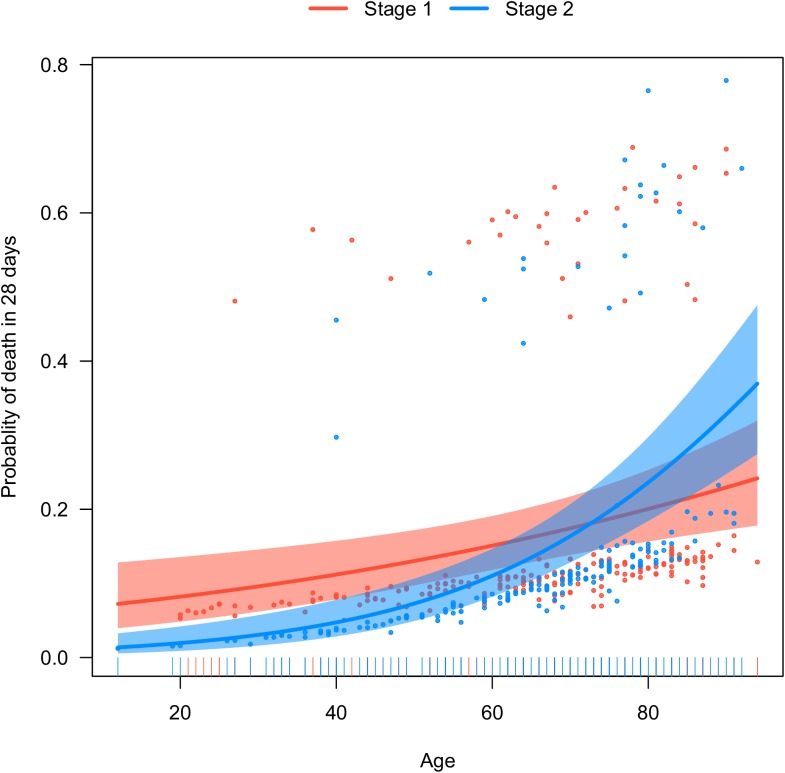
Visualization of the interaction between age and stage. EN feeding protocol tended to benefit younger patients, and was less likely to benefit old ones.

Because there was difference on baseline characteristics between the two stages, multivariable Logistic regression model was employed to adjust for confounding. In the model, the presence of sepsis was independently associated with 28-day mortality (OR: 2.20; 95% CI: 1.02–4.72, Table 6). However, the stage 2 tended to have better mortality outcome than that in stage 1, though the statistical significance was not reached (OR: 0.12, 95% CI: 0.004–2.68). Fig 6 shows the interaction between age and stage. EN feeding protocol tended to benefit younger patients, and was less likely to benefit old ones (Fig 6).

**Table 6:** Multivariable Logistic regression model showing independent predictors of 28-day mortalit.

variables	Odds ratio	lower	upper	P value
Sex (female as reference)	1.33	0.69	2.65	0.403
From emergency	0.58	0.23	1.29	0.202
AGI III	0.35	0.05	1.51	0.205
AGI I	1.18	0.57	2.63	0.661
Parenteral nutrition on day 1	0.77	0.33	1.66	0.518
Age	1.02	0.99	1.05	0.225
Stage 2 versus stage 1	0.12	0.004	2.68	0.196
NRS2002	0.86	0.60	1.19	0.376
Maximum glocose	1.03	0.95	1.10	0.491
Malnutrition	1.03	0.55	1.94	0.923
Sepsis	2.20	1.02	4.72	0.048
AKI	1.82	0.85	3.73	0.111
APACHEII	1.02	0.97	1.07	0.411
SOFA	1.04	0.94	1.14	0.424
Age*Stage	1.03	0.99	1.08	0.190

Abbreviations: BMI: body mass index; NRS: nutrition risk score; EN: enteral nutrition; APACHEII: Acute Physiology and Chronic Health Evaluation II; SOFA: sequential organ failure assessment.

## Discussion

The study found that EN feeding protocol was able to increase the proportion of EN feeding, but failed to reduce 28-day mortality, incidence of nosocomial infection or duration of MV. In exploratory analysis by adjusting for confounding, EN feeding protocol tended to benefit more for young patients than old ones. However, this exploratory analysis did not reach a statistical significance, and thus can only be considered as hypothesis-generating at best.

Consistent with our findings, Padar M and colleagues also found that nurse-driven EN feeding protocol was able to increase EN delivery but failed to reduce mortality rate [19]. In pediatric patients, compliance to feeding protocol was able to reduce time spent without nutrition [20]. With respect to the use of PN, the EN feeding protocol was effective in reducing its use [31]. Volume-based enteral feeding is a feeding strategy that 24-hour calorie goal is established and hourly adjustment was implemented to compensate for feeding interruption. Previous studies have found that this strategy was able to increase the amount of EN delivery [32,33]. However, this strategy was labor-intense that were not feasible in our participating centers. This volume-based protocol was still not able to reduce patient-important outcomes such as mortality, ICU length of stay and duration of MV [34]. Due to its complexity in implementation, such volume-based strategy is not routinely recommended.

Our study found that more EN delivery was not able to reduce mortality. The result is not surprising because several randomized controlled trials have confirmed that full enteral feeding as compared with underfeeding was not beneficial with respect to the mortality outcome [35–37]. In a systematic review and meta-analysis involving 2432 patients, Stuani Franzosi O and colleagues found that the mortality rate was comparable between underfeeding and full feeding groups (RR: 0.91, 95% 0.78–1.06) [38]. An interesting finding in the subgroup analysis was that moderate feeding, which was defined as 46–72% of the total target, was associated with lower risk of death (RR: 0.82; 95% CI, 0.68–0.98) [2]. In our study, the EN feeding proportion was moderate from day 2 to 7 (Table 5). Although there were differences in EN proportion between stage 1 and 2, the absolute difference was 10% at best. Such a small difference may account for the insignificant results of the study. However, There are several trials suggesting that proper implementation of enteral nutritional feeding protocols was able to reduce septic morbidity, ICU/hospital length of stay, mechanical ventilation, and mortality [39–42]. However, due to observational nature of these studies, the impact of enteral feeding protocol on clinical outcomes require further studies to refute or validate. Probably, the protocol can benefit a certain subgroup of patients. Exploratory studies utilizing electronic healthcare records can be employed to identify such a subgroup or certain interaction [43], which is then subject to testing by experimental trials [44].

Several limitations of the study must be acknowledged. This was a before-and-after study. Therefore, confounding factors cannot be fully controlled, especially for those unmeasured confounding factors. We tried to control confounding factors with multivariable analysis, and the result was consistent with that obtained from unadjusted analysis. Missing value was generated, which may compromise the quality of the study. One approach to deal with such a problem is to use complete case analysis. However, the method will result in information loss. In the study, we used single imputation for variables with missing values.

In conclusion, the study found that the EN feeding protocol was able to increase the proportion of EN feeding, but failed to reduce 28-day mortality, incidence of nosocomial infection or duration of MV. In exploratory analysis adjusting for confounding factors, EN feeding protocol tended to benefit more for young patients than old ones. The latter finding is novel and requires further hypothesis-driven studies to ascertain the result.

## Supporting information

S1 Table STROBE checklist. (DOC)

S2 Table De-identified patient data used for current analysis. (CSV)
